# Knowledge and Attitudes for the Management of Behavioral Variant of Frontotemporal Dementia

**DOI:** 10.3389/fneur.2021.786448

**Published:** 2022-01-11

**Authors:** Sheila Castro-Suarez, Erik Guevara-Silva, César Caparó-Zamalloa, Victor Osorio-Marcatinco, Maria Meza-Vega, Bruce Miller, Mario Cornejo-Olivas

**Affiliations:** ^1^CBI en Demencias y Enfermedades Desmielinizantes del Sistema Nervioso, Instituto Nacional de Ciencias Neurológicas, Lima, Peru; ^2^Department of Neurology, Global Brain Health Institute, University of California, San Francisco, San Francisco, CA, United States; ^3^Department of Neurology, Memory and Aging Center, San Francisco School of Medicine, Weill Institute for Neurosciences, University of California, San Francisco, San Francisco, CA, United States; ^4^Neurogenetics Research Center, Instituto Nacional de Ciencias Neurológicas, Lima, Peru; ^5^Center for Global Health, Universidad Peruana Cayetano Heredia, Lima, Peru

**Keywords:** attitude, bvFTD, frontotemporal dementia (FTD), health knowledge, practice

## Abstract

**Background:** The diagnosis of the behavioral variant of frontotemporal dementia (bvFTD) can be especially challenging and is relatively underdiagnosed. There is scarce information on training and attitudes from care providers facing bvFTD in settings with limited resources. We aim to describe clinical knowledge and attitudes facing bvFTD from neurologists, psychiatrists, and residents in Peru.

**Methods:** Potential participants received invitations by email to complete an online questionnaire. In addition, we reviewed 21 curricula from undergraduate medical schools' programs offered by the main schools of medicine in Peru during 2020 and 2021.

**Results:** A total of 145 participants completed the survey. The responders were neurologists (51%), psychiatrists (25%), and residents in neurology or psychiatry (24%). Only 26% of the respondents acknowledged receiving at least one class on bvFTD in undergraduate medical training, but 66.6% received at least some training during postgraduate study. Participants identified isolated supportive symptoms for bvFTD; however, only 25% identified the possible criteria and 18% the probable bvFTD criteria. They identified MoCA in 44% and Frontal Assessment Battery (39%) as the most frequently used screening test to assess bvFTD patients. Memantine and Acetylcholinesterase inhibitors were incorrectly indicated by 40.8% of participants. Seventy six percentage of participants indicated that they did not provide education and support to the caregiver. The dementia topic was available on 95.2%, but FTD in only 19%.

**Conclusion:** Neuropsychiatry medical specialists in Peru receive limited training in FTD. Their clinical attitudes for treating bvFTD require appropriate training focused on diagnostic criteria, assessment tools, and pharmacological and non-pharmacological management.

## Introduction

Frontotemporal dementia (FTD) is considered the second most common cause of dementia between 45 and 64 years (1). FTD is a clinical syndrome caused by degeneration of the frontal and anterior temporal lobes and clinical manifestations include behavioral disturbances, language and executive dysfunction, and sometimes motor symptoms. The main FTD subtypes are behavioral variant frontotemporal dementia (bvFTD), non-fluent/agrammatic variant of primary progressive aphasia, and semantic variant of primary progressive aphasia (2). The complexity of the pathological substrate of FTD is shared by other overlapping degenerative disorders including corticobasal degeneration, progressive supranuclear palsy and amyotrophic lateral sclerosis (3).

Behavioral variant frontotemporal dementia is the most common clinical variant in the FTD spectrum. The presentation age varies from 21 to 85 years of age at onset (4). Clinical discriminating features include early behavioral disinhibition, apathy or inertia, loss of sympathy or empathy, perseverative, stereotyped or compulsive/ ritualistic behavior, hyperorality/dietary changes and dysexecutive neuropsychological profile (5). Psychiatric symptoms are also part of the clinical performance of these patients (6).

Diagnosis of bvFTD can be especially challenging and this entity is relatively underdiagnosed. Some barriers that difficult the diagnosis are: behavioral disturbances in bvFTD can mimic primary psychiatric disorders, validated tools for neuropsychological and social cognition are rarely used in clinical practice, symptoms may be interpreted differently in different cultures and bvFTD is not included as part of medical or residency training (7, 8). Disparities across regions and a little knowledge among health care professionals do not allow timely and accurate diagnosis of bvFTD, which can significantly impact the patients' quality of life and their caregivers' lives, and hinder the development of effective disease-modifying drug treatments (9–11).

Peru is a middle-income country located on the western side of South America on the Pacific Ocean. Peru has about 30 million inhabitants, most of them with mixed ethnicities and an Amerindian ancestry predominance (12). Lima, the capital city, is located on the central coast of the country, and hosts one third of the country's population within it. Most of the largest hospitals and all seven national specialized healthcare institutes in the country are in Lima. Education in Peru is highly unequal, with an overage adult literacy rate of 5.6%^1^. The healthcare system in Peru is administered by five different subsystems, and includes the Ministry of Health (60%), EsSalud for employees (30%), Police and Army forces and private clinics^2^. Since 2020, allPeruvians have been offered a free basic health insurance, called SIS (*Seguro integral de Salud*), for who not documenting other health insurance. There are about 348 neurologists, 78 neurology residents and 690 psychiatrists currently registered in the Peruvian Society of Neurology, the Peruvian Consortium of residents in Neurology and the Peruvian Psychiatric Association. Most of these healthcare professionals work in the capital city. There are 19 neurology residency programs and 18 psychiatry residency programs offered by a total of 10 universities across the country^3^.

We aim to describe the clinical knowledge and attitudes facing bvFTD from neurologists' and psychiatrists as well as the level of undergraduate training in bvFTD in Peru.

## Materials and Methods

### Subjects

Both residents and specialists in neurology and psychiatry from the main cities of Peru were invited to participate in the study. The approach to participants included personal invitations as well as invitations through the Peruvian Society of Neurology, the Peruvian Consortium of Residents in Neurology, and the Peruvian Psychiatry Association. All potential participants were invited to voluntarily participate in the study by an email that contained a description of the study. Participants gave their informed consent by checking on the “yes” button when they decided to participate in the study and filled out an anonymous online questionnaire. The online questionnaire was accessible for a 3-month period (from April to Jun 2021).

We also used a Snowball or Chain-referral sampling method, a non-probability sample technique, in which existing subjects provided referrals to recruit samples for the study. Therefore, the primary data source was the database of the Peruvian Society of Neurology, the Peruvian Consortium of Residents in Neurology, and the Peruvian Psychiatry Association, which nominated other potential data sources that were able to participate in the study.

### The Online-Questionnaire

The online questionnaire was developed by the authors based on current clinical and diagnostic criteria of bvFTD. The content is divided into four main sections: (1) background information, including gender, current work, and medical specialty/ roles at work. (2) Clinical practice related information, including whether they had studied “bvFTD” as part of their medical training, postgraduate education, or had heard or learned about the topic in scientific events or via their own interests. We also added questions regarding the diagnosis or referral of bvFTD cases. (3) Knowledge of diagnosis of bvFTD, including age at onset, supporting symptoms, diagnostic criteria, ancillary lab testing and the neuropsychological patterns of bvFTD, and associated diseases. And (4) Treatment and care of bvFTD patients, including pharmacological and non-pharmacological approaches, caregivers support and palliative care.

The initial questionnaire was further reviewed by six clinicians with expertise on dementias (5 neurologists and 1 geriatrician) for internal validation. A pilot test with the revised version of the questionnaire was performed by 3 health-care professional and trainees (1 medical student, 1 general physician and 1 cardiovascular surgeon) improving unclear phrases. The final questionnaire has a total of 25 multiple-choice questions and needed ~8 min to be completed.

### Research on Schools of Medicine: Syllabi

We gathered available curricula for internal medicine (neurology unit) offered by the main schools of medicine in Peru during the 2020 and 2021 period, and then contacted former neurology residents from many hospitals and medical student scientific societies from different public and private universities in Peru to provide us with the syllabi available to them. Each syllabus was extensively reviewed looking for topics related to frontotemporal dementia.

### Data Analysis

Demographics and main variables were summarized by frequencies and percentages for each multiple-choice question and presented in tables and bar-graphs. To explore differences among neurology and psychiatry subgroups we used chi-square of Fisher exact test (*p* < 0.05, for statistical significance). Statistical analysis was performed on Stata v16.

## Results

**Figure 1:**
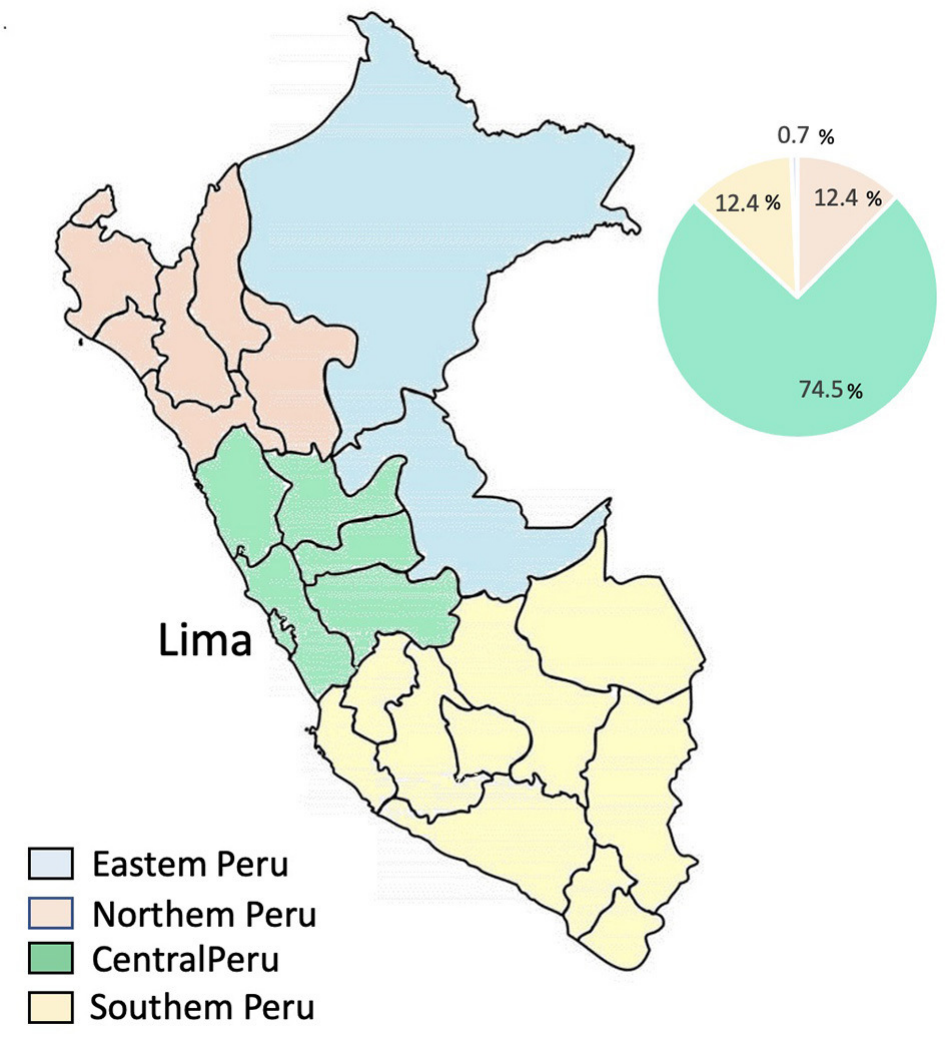
Specialists by region of Peru.

A total of 145 participants (40% female) completed the survey. Most participants were neurologists (51%), followed by psychiatrists (25%), residents in neurology (13%) and residents in psychiatry (11%). The vast majority (75%) of them work in public institutions (73%) in Lima (Figure 1).

Overall, only 26% of the respondents acknowledged receiving at least one class on bvFTD as part of their medical undergraduate training. During residency or other postgraduate training, 66.6% of participants acknowledge classes on bvFTD, and these were mostly in the neurology subgroup (51.38% compared to 15.28%). Similarly, neurologists mostly reported hearing about bvFTD in scientific events like courses, conferences, symposia, or workshops (Table 1).

**Table 1:** Academic profile across specialty.

**Academic profile item**		**Neurologists (*n* = 93)**	**Psychiatrists (*n* = 52)**	*p*-value*
Heard of bvFTD during pre-graduate	Yes	23	15	0.318
	No	56	25	
	I don't remember	14	12	
Heard of bvFTD during post-graduate	Yes	74	22	<0.001
	No	19	29	
Heard of bvFTD during scientific events	Yes	67	18	<0.001
	No	26	29	
Read about bvFTD out of self-interest	Yes	81	32	0.001
	No	12	19	
Specialist time	Residents	21	16	0.128
	<5 years	21	18	
	5–10 years	17	7	
	>5 years	34	11	

*Chi-square.

Regarding experience on clinical practices, 89% of respondents managed at least one dementia case over the past 5 years and about 50% of them managed 1 to 5 bvFTD cases in the same period.

When asked which behavioral/cognitive symptoms must be present to meet the criteria of bvFTD (several signs and symptoms were shown) they (Neurologist and psychiatrist) identified behavioral disinhibition in 91% (*p* = 0.032), followed by early perseverative, stereotyped or compulsive/ritualistic behavior symptoms in 67% (*p* = 0.511). (The two least identified symptoms by respondents) were early apathy or inertia and executive/generation deficits (56%). Despite this, only 25% identified the possible bvFTD criteria (three of six the behavioral/cognitive symptoms must be present to meet the criteria). Probable bvFTD criteria were identified by 18% of the respondents. Histopathological evidence of frontotemporal lobar degeneration was considered as part of the criteria for probable bvFTD by 9% of the participants.

Table 2 reveals the top-ranked most-related disorders when considering distinct clinical phenotypes associated with multiple neuropathologic entities of bvFTD. Forty five percentage of the participants indicated that Corticobasal degeneration is the most relevant disease related to bvFTD, followed by motor neuron disease and progressive supranuclear palsy. Neurologists identified those diseases better than psychiatrists; these results were statistically significant. However, the participants identified Lewy Body Disease 56 (40%) and Parkinson's disease 37 (27%) as related disorders associated with multiple neuropathologic entities of bvFTD.

**Table 2:** Clinical phenotypes associated with multiple neuropathologic entities of bvFTD.

	**Neurologists *n* = 93**	**Psychiatrists *n* = 52**	**Total *n* (%)**	*p*-value*
Corticobasal degeneration (CBD)	47	15	62 (44.6)	0.011
Lewy body dementia (LBD)	35	21	56 (40.3)	0.744
Motor neuron disease (MND)	41	13	54 (38.8)	0.023
Progressive supranuclear palsy (PSP)	35	11	46 (33.1)	0.041
Parkinson's disease (PD)	18	19	37 (26.6)	0.023
Multiple system atrophy (MSA)	22	6	28 (20.1)	0.076

*Chi-square.

The professionals checked all screening test options they usually employ to assess bvFTD patients. The Montreal cognitive assessment (MoCA), Frontal Assessment Battery (FAB), Mini-mental state examination (MMSE) and INECO frontal screening (IFS) were the most widely used tests with 44, 39, 38, and 36%, respectively.

The participants checked all treatment options they usually recommend for patients with bvFTD diagnoses. Memantine and Acetylcholinesterase inhibitors are indicated by 40.8% of participants who diagnose and treat bvFTD. Selective serotonin reuptake inhibitors (39.4%), other pharmacological (gingko biloba, antipsychotics) and non-pharmacological treatment (cognitive rehabilitation) that are usually indicated were (7%). Besides, 12.7% of responders in this study answered that there is not a pharmacological treatment for the patients.

We asked about strategies that maximize comfort for bvFTD patients and their families, such as information, psychosocial support, and caregiver education. The vast majority of participants (76%) indicated that they do not provide education, information, and support to the caregiver of the bvFTD patient. Of the group that provides this type of support (24%), 91.3% do so as part of their outpatient consultation. Participants reported that 88% of patients with advanced bvFTD were not followed by a palliative care team.

**Figure 2:**
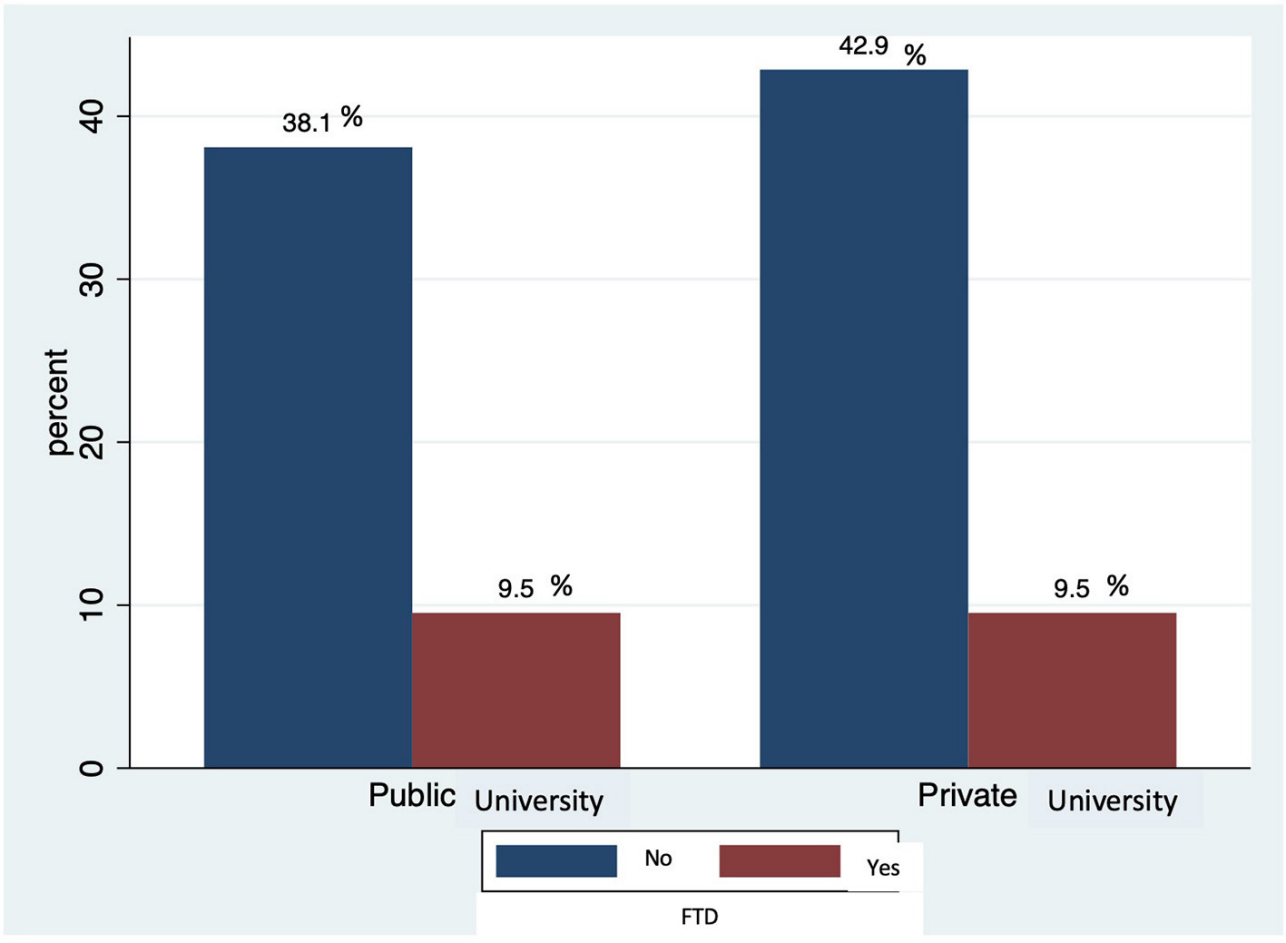
Topical of Frontotemporal dementia (FTD) by medical school category.

We reviewed 21 curricula from 34 different undergraduate medical schools' programs between 2020 and 2021. The dementia topic was available on 95.2% of them and the frontotemporal dementia topic was specifically described in 19% of them, specifically the behavioral variant of frontotemporal dementia was not mentioned (Figure 2).

## Discussion

Based on the online-survey results, most of the participants declared having limited training activities related to behavioral variant frontotemporal dementia both as undergraduate students and as postgraduate residents in neurology or psychiatry. There are several social, economic, and educational factors that might be affecting proper FTD training in clinicians in developing countries such as Peru.

Lower rates of training activities in undergraduate medical programs might affect the quality of management of FTD in Peru. The lower training levels in FTD (19%) in medical school, is not only explained by formal curricula, but also by low awareness of the impact of dementia and its implications for health and the economy. None of undergraduate medical school curricula formally includes specific topics for bvFTD, as these are mostly focused on Alzheimer's disease and vascular cognitive decline, neglecting other causes of dementia. This contrasts with curricula from European and North-American countries (13). Despite low rates of formal training, early exposure of medical students during clinical rotations, which was not systematically addressed on our survey, and this might positively increase the medical skills for working with these dementias. Considering that most of these specialists are concentrated in the Capital city and other main cities of the country, it is quite possible that primary care physicians are the first point of contact by FTD patients. Therefore, primary care providers must be adequately trained to identify or suspect FTD and refer these cases for better evaluations at bigger institutions. As a result, changes in undergraduate and graduate curricula in accredited medical schools in our country is warranted.

About of half of the participants declared having been trained in bvFTD during neurology or psychiatry residency program, consistent with a previous Peruvian survey performed in 2017 (14), but this is much lower than overall Latin American reporting 86 to 96% postgraduate training rates (7). Progressive harmonization of postgraduate medical curricula among universities, just as has happened for undergraduate programs, are required to improve the current situation. In addition, it will be important to conduct studies that explore the factors that influence the learning process of future health professionals.

The vast majority of participants are unclear or unfamiliar with the diagnostic criteria for possible and probable bvFTD. We observed, that clinicians may identify isolated behavioral/cognitive supportive symptoms for bvFTD; however, they fail to recognize the minimum number of symptoms required for considering a possible bvFTD diagnosis (5). Diagnostic workup turns more complicated since neuroimaging supporting features (MRI, CT, hypoperfusion or hypometabolism on PET or SPECT) are also required for considering probable bvFTD (5). There is a clear preference of respondents to use the most common screening cognitive assessment tools, like MMSE and MoCA, over more specific tests with higher diagnostic index for FTD. The MMSE and MoCA are screening cognitive assessment tools that do not differentiate FTD from other dementias (15). There are validated cognitive tests with over 90% of specificity for bvFTD, such as the Adddenbrooke's Cognitive examination (ACE) (15) and IFS (16). Both theoretical conferences and case-scenario workshops are needed to improve clinical competencies for FTD diagnosis. Disseminating and promoting the use of validated assessment tools in our population for bvFTD patients will be an essential task in the coming years, and involving the universities, neurology and psychiatry associations could prove strategic for achieving this goal.

The low identification rate of FTD will affect early diagnosis and management of these disorders in clinical practice. We found <50% of the participants identified motor neuron disease (MND), progressive supranuclear palsy (PSP) and corticobasal degeneration (CBD) as FTD related disorders. Therefore, patients experiencing combined symptoms might be even more challenging for early diagnosis. The identification of overlapping syndromes is important because it helps predict tau-positive pathology from a CBD- or PSP-like presentation, whereas frontotemporal dementia syndrome and MND almost certainly predicts TDP-43 pathology (17, 18). It is noteworthy that 26–40% identified synucleinopathies like dementia with Lewy bodies and Parkinson's disease (PD) as FTD related disorders, reflecting the large number of scientific activities related to PD and related disorders.

Most of the respondents declare that they manage bvFTD based on pharmacological-only management and are mostly focused on AD-related medication. Memantine and Acetylcholinesterase inhibitors were the most common drugs used for treating bvFTD, consistent with previous reports from Latin American countries (7). This relatively common practice might negatively affect bvFTD, since memantine (NMDA antagonist) and cholinesterase inhibitors failed to improve behavior and worsened cognition in patients with bvFTD in several studies (18, 19). Only 40% of respondents identified Selective serotonin reuptake inhibitors (SSIRs) as a pharmacological treatment option for patients, which is in accordance to current recommendations highlighting SSRIs for control FTD behavior abnormalities (18). Very few participants (<10%) considered non-pharmacological strategies as part of bvFTD management. Coordinated work between affected patients and caregivers, including regular personalized activities that prevent abnormal behaviors, among other strategies, are strongly recommended as part of global and multidisciplinary care (20). Despite all these features, most respondents declared an interest in receiving training on diagnosis and management of bvFTD, and, as such, training programs must be implemented to address these gaps. A multidisciplinary approach is strongly recommended for involving care providers, patients and their families, caregivers, and patient's associations to improve the patient's quality of life.

Survey respondents do not regularly educate caregivers on disease-related aspects nor on end-of-life care. It is fundamental that the caregivers receive information and training on complex medical symptoms, psychosocial issues, spiritual well-being, and planning for the future (21). This interdisciplinary approach to care improves the quality of life and reduces the suffering of the patients and their families. In addition, discussions on identifying signs of distress, anxiety or depression in caregivers would allow them to prevent or manage these disorders earlier (22). The fact that not having caregiver-oriented programs focused on bvFTD in Peru might also affect caregiver education. Palliative care approaches are quite limited among respondents; this is probably related to the scarcity of palliative care programs in Peru, which mainly focus on oncological pathology (23). Establishing palliative care programs in institutions could also improve health outcomes and lower costs when taking care of these patients.

We recognize the limitations of the study and that only including neurologists and psychiatrists that mostly work in the capital city may mean that there is an overestimation of some training and management aspects that are likely much more difficult to address in remote regions of the country. The survey used for this study has been elaborated by authors with further review and feedback by experts in the field, but is not validated, then it might be possible that some of the answers might be biased. A more representative sample of health care providers from different specialties and from diverse regions together with a more robust survey model designs might better explain some of the gaps found in this survey.

In conclusion, neurology and psychiatry residents and specialists in Peru receive limited training in FTD. Training programs should focus on diagnostic criteria, assessment tools and pharmacological and non-pharmacological management, and palliative care.

## Data Availability Statement

The data supporting the conclusions of this article are available from the corresponding author, SC-S, upon reasonable request.

## Ethics Statement

This study involves human participants and was reviewed and approved by Institutional Ethics Research Committee at Instituto Nacional de Ciencias Neurologicas (Cert. N 013-2021 CIEI-INCN). Written informed consent for participation was not required for this study in accordance with the national legislation and the institutional IRB requirements.

## Author Contributions

SC-S, EG-S, and MC-O conceptualized and designed the study, data collection, performed statistical analyses, and drafted the manuscript. CC-Z and VO-M designed the study and drafted the manuscript and data collection, MM-V and BM designed the study and drafted the manuscript. All authors took part in critical revision of the manuscript and approved the submitted version.

## Funding

The study was partially supported by Instituto Nacional de Ciencias Neurologicas. This work was supported in part by the MULTI-PARTNER CONSORTIUM TO EXPAND DEMENTIA RESEARCH IN LATIN AMERICA (ReDLat), with funding from the National Institutes of Aging of the National Institutes of Health Under Award Number R01AG057234, an Alzheimer's Association Grant (SG-20-725707-ReDLat), the Rainwater Foundation, and the Global Brain Health Institute.

## Author Disclaimer

The content is solely the responsibility of the authors and does not represent the official views of the National Institutes of Health, Alzheimer's Association, Rainwater Charitable Foundation, or Global Brain Health Institute.

## Conflict of Interest

The authors declare that the research was conducted in the absence of any commercial or financial relationships that could be construed as a potential conflict of interest.

## Publisher's Note

All claims expressed in this article are solely those of the authors and do not necessarily represent those of their affiliated organizations, or those of the publisher, the editors and the reviewers. Any product that may be evaluated in this article, or claim that may be made by its manufacturer, is not guaranteed or endorsed by the publisher.

## Footnotes

^1^ https://www.macrotrends.net/countries/PER/peru/literacy-rate

^2^ https://www.who.int/workforcealliance/countries/per/en/

^3^ https://www.conareme.org.pe/web/proceso-de-admision-2021.php
